# Tumor grafts grown on the chicken chorioallantoic membrane are distinctively characterized by MRI under functional gas challenge

**DOI:** 10.1038/s41598-020-64290-z

**Published:** 2020-05-05

**Authors:** Conny F. Waschkies, Fatma Kivrak Pfiffner, Dorothea M. Heuberger, Marcel A. Schneider, Yinghua Tian, Petra Wolint, Maurizio Calcagni, Pietro Giovanoli, Johanna Buschmann

**Affiliations:** 1 Center for Surgical Research, University Hospital Zurich, Zurich, Switzerland; 2 Institute of Medical Molecular Genetics, University of Zurich, Zurich, Switzerland; 3 Institute of Intensive Care Medicine, University Hospital Zurich, Zurich, Switzerland; 4 Visceral and Transplant Surgery, University Hospital Zurich, Zurich, Switzerland; 5 Plastic Surgery and Hand Surgery, University Hospital Zurich, Zurich, Switzerland

**Keywords:** Cells, Solid-state NMR

## Abstract

Recently, a tumor model based on the chorioallantoic membrane (CAM) was characterized structurally with Magnetic Resonance Imaging (MRI). Yet, capability of MRI to assess vascular functional reserve and potential of oxygenation-sensitive MRI remain largely unexplored in this model. For this purpose, we compared MC-38 colon and A549 lung adenocarcinoma cell grafts grown on the CAM, using quantitative T1 and T2* MRI readouts as imaging markers. These are associated with vascular functionality and oxygenation status when compared between periods of air and carbogen exposure. Our data show that in A549 lung adenocarcinoma cell grafts T2* values increased significantly upon carbogen exposure (p < 0.004, Wilcoxon test; no change in T1), while MC-38 grafts displayed no changes in T1 and T2*), indicating that the grafts differ in their vascular response. Heterogeneity with regard to T1 and T2* distribution within the grafts was noted. MC-38 grafts displayed larger T1 and T2* in the graft centre, while in A549 they were distributed more towards the graft surface. Finally, qualitative assessment of gadolinium-enhancement suggests that A549 grafts display more prominent enhancement compared to MC-38 grafts. Furthermore, MC-38 grafts had 65% larger volumes than A549 grafts. Histology revealed distinct underlying phenotypes of the two tumor grafts, pertaining to the proliferative status (Ki-67) and cellularity (H&E). In sum, a functional gas challenge with carbogen is feasible through gas exchange on the CAM, and it affects MRI signals associated with vascular reactivity and oxygenation status of the tumor graft planted on the CAM. Different grafts based on A549 lung adenocarcinoma and MC-38 colon carcinoma cell lines, respectively, display distinct phenotypes that can be distinguished and characterized non-invasively *in ovo* using MRI in the living chicken embryo.

## Introduction

The chorioallantoic membrane (CAM) of the developing chicken embryo is an established model that is used in biomedical research in a multitude of different applications^1^. For instance, it is employed in screening biomaterials^2–4^, testing microsurgical procedures^5^, drug delivery systems and biosensors^6,7^, and in toxicity and pharmacokinetic studies^8,9^. Recently, the CAM model was used to asses perfusion capacities of on-planted biomaterials with Magnetic Resonance Imaging (MRI) as a non-destructive imaging readout^10^.

The CAM serves as a support for the respiratory capillaries outside the embryo. It is highly vascularized and allows for gas exchange between the embryo and its environment. This renders the CAM a suitable model to study angiogenesis^11–14^. Notably, as a natural immunodeficient host with a rich vascular network, the CAM is particularly capable to sustain grafted tissues and implants for tissue engineering applications^15^. Most importantly, it provides an advantageous environment for tumor formation and is therefore often used to study tumor development, metastasis and progression in xenotransplanted tumors^16^. Another advantage is the easy access to the CAM through a window in the eggshell, which allows for continuous visualization of the implant/graft site. Finally, the CAM model serves as an intermediate step between cell culture studies and more complex mammalian *in vivo* models. Taken together, the CAM model represents a simple to maintain, rapid, low-cost assay for a multitude of different biomedical applications.

Recently, tumor growth on the chicken CAM was monitored and structurally characterized with MRI *in ovo*^17,18^. Vascular functional reserve and oxygenation-sensitive MRI measures^19,20^, however, remain largely unexplored in this model. The exploration of these factors will lead to an improved understanding of tumor vascular development and maturation, tumor heterogeneity and hypoxia from an MR imaging perspective.

Oxygen tension in (tumor) tissue is fundamentally determined by the balance between oxygen availability and consumption. Oxygen availability is dictated by the supply through blood flow and microvascular O2 concentration, which in turn is determined by perfusion pressure, arterial partial pressure of oxygen (pO2) and relative oxygenation saturation of haemoglobin (Hb/HbO2 ratio), among others (haematocrit, drugs etc.). Oxygen consumption is assumed to remain unchanged under hypercapnic and hyperoxic conditions.

Tissue oxygenation status can be assessed non-invasively with T2*-sensitive MRI (blood oxygenation level dependent, BOLD MRI^21^) and oxygen-enhanced (OE) MRI. T2*-sensitive MRI is based on the magnetic properties of haemoglobin which is paramagnetic in its de-oxygenated form and diamagnetic in its oxygenated form. T2* based MRI is hence sensitive to the relative Hb/HbO2 ratio in vessels, but also to blood volume, and therefore is not uniquely related to oxygenation status. However, changes in tumor oxygenation can be qualitatively assessed during carbogen breathing^22–24^.

OE MRI is based on the effect that dissolved oxygen in blood acts as a T1-shortening paramagnetic contrast agent. It may detect presence of excess dissolved O2 in plasma upon a hyperoxic challenge. Comprehensive studies suggested that in normoxic tissues in the presence of fully oxygenated haemoglobin such excess O2 is dissolved in the blood pool, shortening T1. In hypoxic tissues, however, excess oxygen is bound to haemoglobin to replenish blood oxygen stores, going along with negligible O2 dissolution in plasma and therefore no change in T1^20,25,26^.

Vasoreactivity is the capacity of the vasculature for vasodilation and is typically assessed as a functional stress test, measuring changes in perfusion in response to vasoactive stimuli. Often, T2* weighted BOLD MRI is employed in this regard for its technical simplicity, availability and good signal-to-noise ratio (SNR) in clinical settings^27–29^, though it represents only an indirect appraisal for relative perfusion change. Approaches that are more direct include arterial spin labelling or dynamic contrast enhanced MRI.

Non-invasive MR imaging of tumors on the CAM under gas challenge offers a viable option to assess tumor characteristics that may help in the selection of a proper therapeutic approach and to monitor treatment response. For example, tumor hypoxia results from an uncontrolled excessive cell proliferation, often encountered in malignant, aggressive phenotypes and is associated with increased metastasis, uncontrolled angiogenesis and resistance to radio- and chemotherapy. In such tumors, there is a relative oxygen deficit, reflected in high Hb/Hb O2 ratios. The application of a hyperoxic gas challenge can lead to lower Hb/HbO2 ratios, resulting in increased T2* values. In contrast, if normoxic tumors are exposed to hyperoxic conditions, the amount of O2 dissolved in the blood plasma increases, resulting in lower T1 values. Simultaneous vasoconstriction triggered by elevated oxygen exposure represents a competing effect by lowering the blood perfusion.

In the present study with a hypercapnic-hyperoxic gas challenge by carbogen (5% CO2 and 95% O2), we expect that the elevated CO2 level (hypercapnia) triggers vasodilation, leading to an increased blood perfusion and hence to an overall increased oxygenations status. On the other hand side, the high level of O2 (hyperoxia) leads to a lower Hb/HbO2 ratio in the blood, unless haemoglobin is already oxygen-saturated. In that case, excess O2 cannot be bound to haemoglobin, but will increase the dissolved O2 in the blood. Notably, excess O2 leads to a vasoconstriction, which has to be taken into account when discussing the oxygenation status under hyperoxic conditions. Excess O2 in the blood and a simultaneous vasoconstriction may result in an overall constant oxygenation status, evoked by the two competing effects. Taken together, response to hypercapnic hyperoxia (carbogen) in T2*- and T1-sensitive MRI readouts offers a qualitative approach to assess the vasoreactivity of the tumor graft vasculature and helps distinguishing between hypoxic and normoxic tumor tissue.

MC-38 colon and A549 adenocarcinoma cell grafts were selected in the present study, representing murine and human cell lines as well as two different tumor types (colon and lung cancer, respectively). They were grown on the CAM and compared with regard to their vascular and oxygenation phenotypes. For this purpose, quantitative T2* and OE T1 MRI are explored as functional *in vivo* imaging markers when compared between periods of air and hypercapnic-hyperoxia (carbogen) exposure. We demonstrate that a functional gas challenge with carbogen is feasible through the CAM, allowing to access vascular function and oxygenation status of the tumor graft in this experimental model.

## Methods

### CAM assay & cell preparation

For experiments in chicken embryos until embryonic day 14 no IACUC approval is required according to Swiss animal care guidelines (TSchV, Art. 112). Fertilized Lowman white LSL chick eggs (Animalco AG Geflügelzucht, Staufen, Switzerland) were incubated at 37 °C and 65% relative humidity. On incubation day (ID) 3.5, a circular window was excised into the eggshell after removing 2 ml albumen so that the developing CAM detached from the eggshell (Supplementary Information Fig. 1A).

Two cell lines were chosen to generate tumor grafts on the CAM on ID 7: A549 cells (ATCC), a human lung alveolar cancer cell line, as well as MC-38 (Kerafast), a murine colon cancer cell line, syngeneic on a C57BL/6 background (Supplementary Information Table 1). For that purpose, MC-38 cells were cultivated in DMEM (Life Technologies, Zug, Switzerland), supplemented with 10% FBS and 100 U/mL of penicillin and streptomycin, and incubated at 5% CO2 and 37 °C. A549 cells were cultivated in DMEM (Life Technologies), supplemented with NEAA, L-Glutamine and 10% FBS and incubated at 5% CO2 at 37 °C. The cells were harvested with trypsin (0.5%), were centrifuged and resuspended in serum free DMEM. For tumor graft generation, MC-38 cells below passage 7 (P7) and A549 cells below P13 were used. The cell suspension was 1:1 diluted with ice-cold growth factor-reduced matrigel (Corning) to a concentration of 0.5*10^6^ cells/50ul. On a sterile petri dish, droplets of 50ul of the cell-matrigel suspension were formed and pre-warmed for 10 min at 37 °C. One such droplet was added on the CAM by a sterile 1 ml tip in the middle of a 1 cm-diameter plastic ring to flatten the CAM surface and as a landmark to locate the developing tumor grafts (Supplementary Information Fig. 1B). Eggs were further incubated until ID 14.

### Magnetic resonance imaging

On ID 14, vascular response and oxygenation of the A549 and MC-38 carcinoma cell grafts grown on the chicken embryo’s chorioallantoic membrane was studied *in situ* on the CAM (“*in ovo*”) of the living chicken embryo using MRI. For the MRI examination, eggs were placed onto a custom-built sliding bed and enveloped by warm water tubing (37 °C) to maintain the temperature of the chicken embryo in a physiological range. Chicken embryos were sedated with 0.3 mg/kg medetomidine (diluted 1:100, volume 0.3 ml) dripped onto the CAM prior start of MRI examinations, and again immediately prior contrast-enhanced MRI when carried out.

MRI was performed in A549 (n = 14) and MC-38 graft samples (n = 12) on a 4.7 T cm Bruker PharmaScan system (Bruker BioSpin, Ettlingen, Germany), equipped with an actively decoupled two-coil system, consisting of a 72 mm quadrature resonator for excitation and a 20 mm single loop surface coil for reception. Gases were delivered through a plastic tube at 200 ml/min flow rate directed onto the CAM, which serves as a breathing organ during chicken embryo development. To prevent excessive loss of moisture, the egg shell window was kept covered by a sterile plastic plate onto which the surface coil was attached directly above the graft for optimal signal sensitivity and through which the gas tube was guided **(**Supplementary Information Fig. 1B).

MR images were collected from 4 sagittal slices comprising the tumor graft with a FOV of 4.5 × 2.7 cm and a spatial resolution of 300 × 300 um3 (image matrix 150 × 90) and a slice thickness of 800 um with an interslice gap of 200 um. Standard T2w RARE and T1w FLASH acquisitions were obtained for anatomical reference (T1w FLASH acquisition at higher spatial resolution of 150 × 150 um2, image matrix 300 × 300). Relaxometry measurements were performed with a RARE sequence at variable repetition times TR (TR 430/800/1500/3000/4500 ms, RARE factor 2, TE 10 ms, acquisition time 6 min) and a multi-echo gradient-echo (MGE) acquisition (TE 4–81 ms, echo spacing 7 ms, 3 averages, TR 1500 ms, acquisition time 5 min) for quantitative T1 and T2* mapping (referred to as qT1 and qT2*), respectively. qT1 and qT2* relaxation times serve as tissue-dependent markers associated with vascular functionality and oxygenation status when compared between periods of air and carbogen (5% CO2, 95% O2) exposure.

In selected samples T1w anatomical references scans were repeated 15 min after i.v. injection of 100 uL Gd-DOTA (Dotarem, Guerbet S.A., Switzerland) to study contrast enhancement in the graft. For intravenous injection, eggs were placed on a 37 °C heating pad and i.v. injection was performed under a surgical microscope with 12–20 x magnification. A big and straight vein on the surface was selected and grasped by a microsurgical forceps and 100 µL Gd-DTPA slowly injected with a 1.0 mL syringe and 30 G needle. A cotton sticker was placed on the injection site of vessel with slight pressure before the syringe was withdrawn to prevent bleeding.

### Histology and Immunohistochemistry

After completion of the MRI measurements, the graft was fixed overnight using 4% formalin solution in PBS, then excised, embedded in paraffin, cross-sectioned into 5 µm slices and stained for H&E (cellularity) and Ki-67 (proliferation).

For Ki-67 staining, samples were pre-treated in PT Link (DAKO) with Envision Flex Target Retrieval Solution Low pH (DAKO, K8005) and incubated with monoclonal mouse anti human Ki-67 MIB-1 antibody (DAKO, IR626, dilution: RTU) for A549 tumors or with monoclonal rabbit anti mouse Ki-67 (SP6, Abcam, 16667, dilution 1:200 with buffer from DAKO) for MC-38 tumors, respectively. Then, secondary antibody was applied, consisting of labelled Polymer–HRP anti mouse (DAKO, K4007, dilution: RTU) for A549 tumors or labelled Polymer–HRP anti rabbit (DAKO, K4003, dilution: RTU) for MC-38 tumors. After that, staining was performed in an Autostainer Link48 (DAKO), with Flex DAM and Substrate- Chromogen (DAKO, K3468) and Envision Flex Hematoxylin ready to use (DAKO, K8008).

For HIF-1-α staining of both cells grafts (n = 5 randomly selected for A549 and MC-38, respectively), a mouse monoclonal antibody (abcam, ab16066, 1:1000) was used. Briefly, samples were pre-treated in PT Link (DAKO) with Envision Flex Target Retrieval Solution high pH = 9.0 (DAKO, K8004) and then incubated with anti-HIF-1-α for 1 hour. Then, secondary antibody was applied, consisting of labelled EnVision HRP/ mouse (DAKO, K4001, dilution: RTU) for 20 min. After that, staining was performed in an Autostainer Link48 (DAKO), with Flex DAM and Substrate- Chromogen (DAKO, K3468) and Envision Flex Hematoxylin ready to use (DAKO, K8008). For the quantitative determination of HIF-1-α positive cells per area, all cells stained dark brown were counted in the whole cross-section area of the respective tumor graft.

Vessel density was assessed in H&E stained sections based on one section through the middle of the cell graft (n = 5 randomly selected for A549 and MC-38, respectively). Vessels were analyzed according to their morphology and eventual erythrocytes within the lumen. The area of the section was determined with Synedra View software (version 18.0.0.7). The vessel density was counted as number of vessels per area (mm^−2^). Furthermore, staining with two different lectins was used to confirm chicken vessels within the human and murine tumor, respectively. Protocols for lectin stainings included Sigma Aldrich lectin (L0651, batch SLBQ4937V; dilution 1:2) and Vector laboratories lectin (B1305; dilution 1:500). After incubation with corresponding lectins for 1 hour, streptavidin/HRP (DAKO, 1:500) was used. After that, staining was performed in an Autostainer Link48 (DAKO), with Flex DAM and Substrate- Chromogen (DAKO, K3468) and Envision Flex Hematoxylin ready to use (DAKO, K8008).

### Data analysis

Quantitative T1 and T2* maps were computed from RARE acquisitions at multiple TR and MGE acquisitions at different TEs, respectively, by exponential signal fitting (integrated in Brukers Paravision 5.1. MRI acquisition and reconstruction software). Graft response to the stimulus was determined as the change in qT2* and T1 values under the carbogen challenge as compared to the baseline when the chicken embryo was exposed to medical air in a region of interest comprising the graft. Graphs and statistical analyses were produced by R v3.5.0 (R Foundation for Statistical Computing, Vienna, Austria), using a paired-samples Wilcoxon test for testing between conditions (air vs. carbogen) in both graft types. An unpaired t test was used to compare the vessel densities and densities of HIF-1-α positive cells in A549 and MC-38 cell grafts, where the data were normally distributed (Shapiro Wilk test) and the variances were homogene (Levene’s test).

## Results

### Graft anatomical structure and appearance

**Figure 1:**
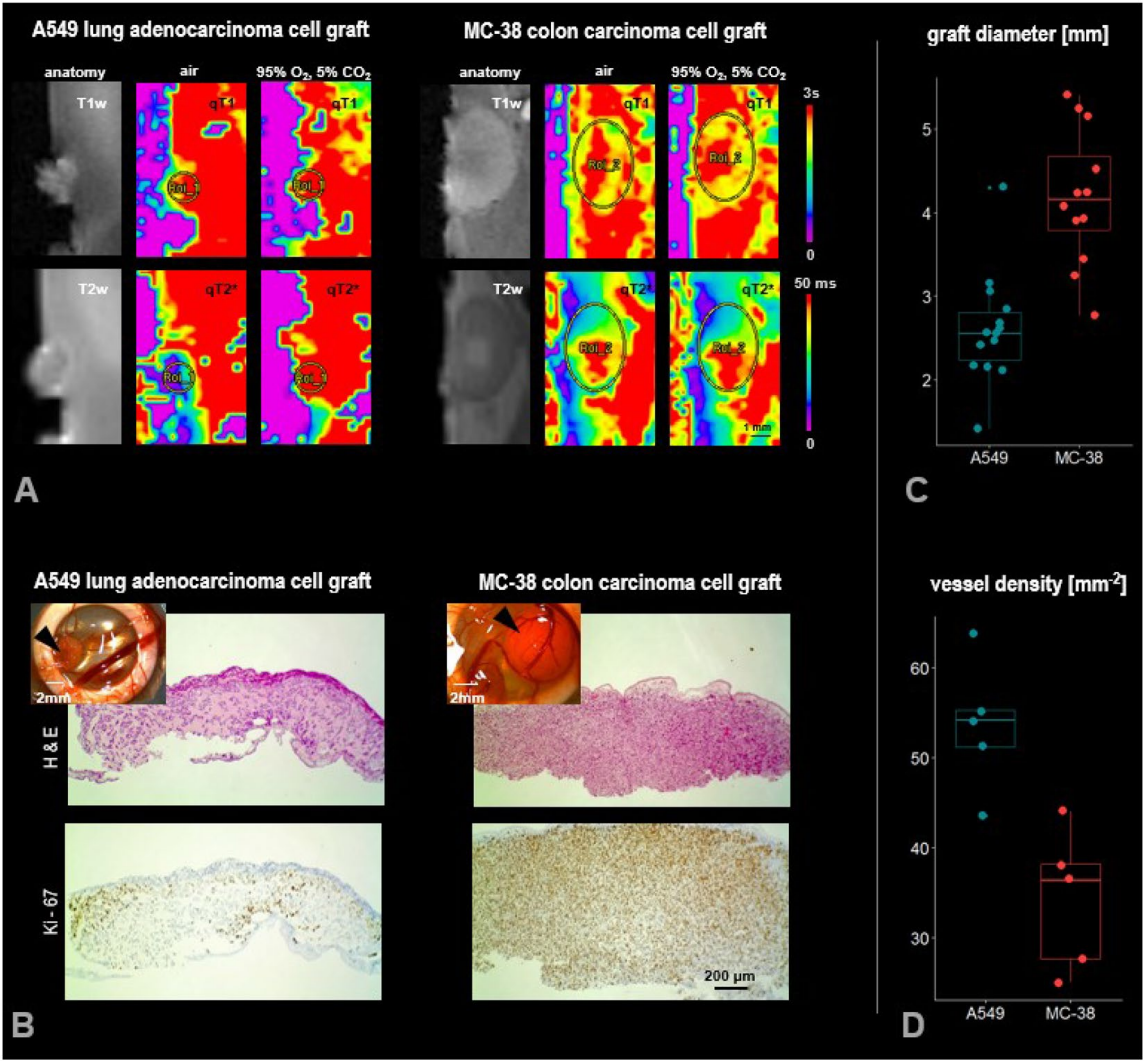
(**A**) *In ovo* MRI images of A549 lung adenocarcinoma and MC-38 colon carcinoma cell grafts grown on the CAM of the chicken embryo for 7 days. Grafts are shown in T1w and T2w anatomical reference images and are outlined with regions of interest on quantitative color-coded T1 (qT1) and T2* (qT2*) maps obtained while the graft was exposed to medical air and carbogen, respectively. (**B**) Comparative histology. Shown are sample slices from both graft types stained for H&E (top) and Ki-67 (bottom), respectively. Inserts: Supportive plastic ring with the graft (arrowhead) on the CAM, photographed after extraction of the CAM. Both graft types display distinct phenotypes with regards to cellularity (H&E) and proliferative status (Ki-67). (**C**) Graft size comparison. Quantitative comparison of graft diameter between A549 lung adenocarcinoma and MC-38 colon carcinoma cell grafts. (**D**) Vessel density comparison. Quantitative comparison of vessel density in A549 and MC-38 cell grafts.

MR images were obtained from all tumor grafts with sufficient SNR and image quality to depict the grafts in T1w and T2w anatomical images. Grafts were as well delineated in the quantitative T1 and T2* images and corresponding color-coded parametric maps obtained during air and carbogen exposure (Fig. 1A). Graft sizes were different, which was obvious in the anatomical images. The MC-38 colon carcinoma cell grafts had a 65% larger graft diameter (4.2 ± 0.8 mm) compared to the A549 adenocarcinoma cell grafts (2.6 ± 0.7 mm, Fig. 1C).

### Response to carbogen exposure

We compared MC-38 colon and A549 lung adenocarcinoma cell grafts using quantitative T1 and T2* MRI readouts. These served as imaging markers associated with vascular functionality and oxygenation status when compared between periods of air (baseline) and carbogen exposure (functional gas challenge, here hypercapnic hyperoxia).

Quantitative parametric T1 and T2* maps show spatial variation in basal T1 and T2* obtained during exposure to air. In MC-38 grafts, T1 values are larger in the graft centre, and the same applies for the T2* values, while the centre of larger T2* values in one probe was shifted laterally. This pattern has been consistently observed in all MC-grafts. In A549 grafts, larger T1 and T2* values were found more distributed towards the graft/CAM surface.

Response to carbogen exposure is accessible in larger T2* values, more prominently observed in A549 grafts and distributed towards the graft periphery. In MC-38 grafts, T2* increase was more ambiguous, with increase observed in six out of twelve samples, affecting mostly the centre of the graft and decrease or no change seen in the other samples. Response to carbogen was furthermore detected in change in T1 with slight decrease in MC-38 grafts (except in one sample), but no consistent change in T1 in A549 grafts (increase in nine samples, decrease in four samples, no change in one sample), respectively, in the parametric maps. Regional heterogeneity in the carbogen response was observed in both graft types. There were increases as well as decreases in T1 within the same grafts (Fig. 1A).

**Figure 2:**
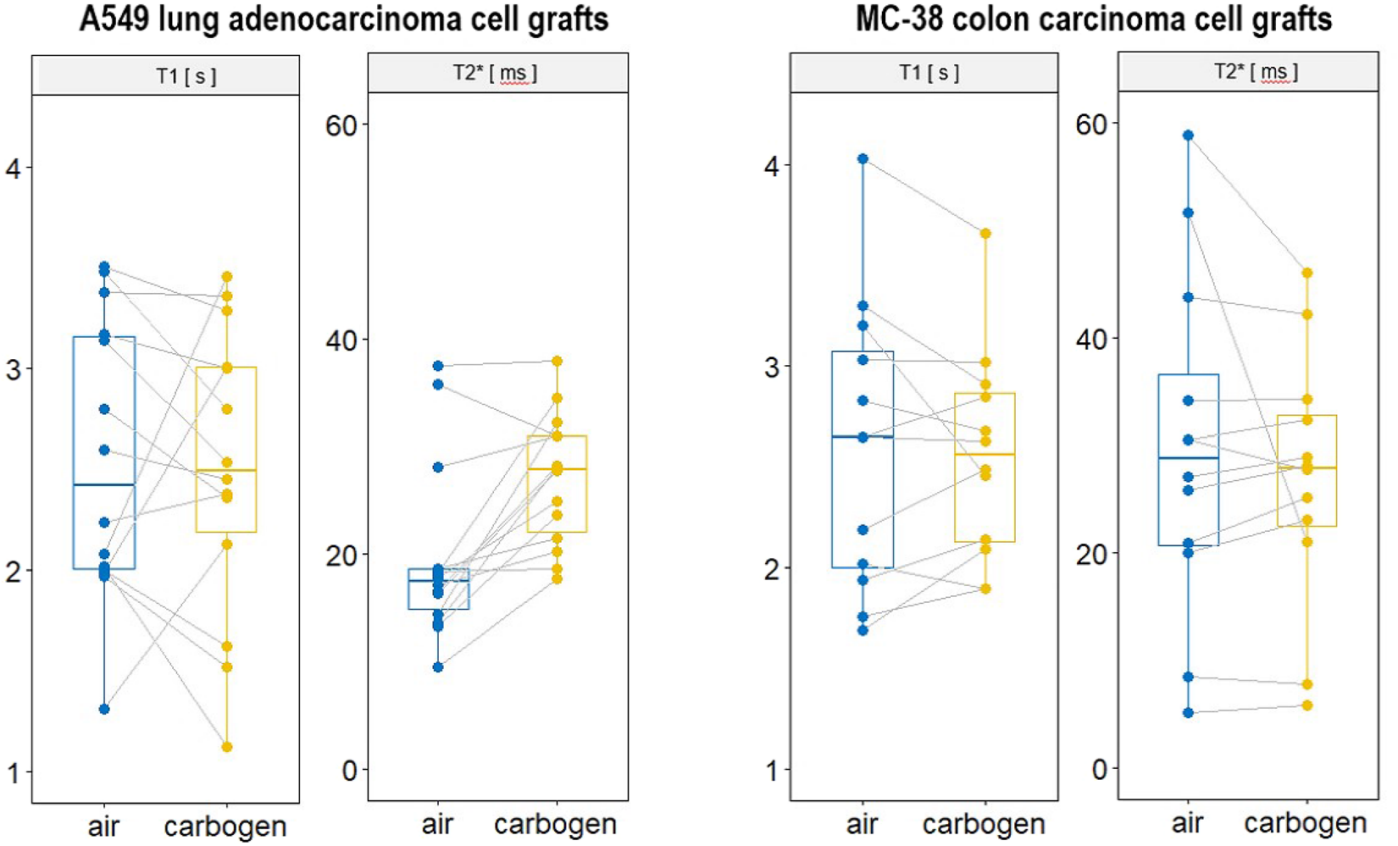
Quantitative analyses of changes in T1 and T2* upon carbogen gas challenge, as compared with medical air, in A549 lung adenocarcinoma cell grafts and in MC-38 colon carcinoma cell grafts, respectively. In A549 lung adenocarcinoma cell grafts, T2* values increased by 37% upon carbogen exposure (p < 0.004, Wilcoxon test) while MC-38 colon carcinoma cell grafts displayed no consistent trends in T1 and T2*.

To compare the two tumor grafts, a graphical approach was performed by region of interest (ROI) analysis comprising the whole graft. Our data show that in A549 lung adenocarcinoma cell grafts T2* values significantly increased by about 37% upon carbogen exposure (p < 0.004, Wilcoxon test; no change in T1, p = 0.45), while MC-38 grafts displayed no consistent trends in T1 (p = 0.84, Wilcoxon test; no change in T2*, p = 0.97, Fig. 2). T1 response in A549 grafts and T2* in MC-38 grafts was more ambiguous, displaying different trends between samples and larger distribution between (ROI-averaged) basal values. Based on HIF-1-α positive cells per area, the density for hypoxic cells was higher for MC-38 grafts compared with A549 grafts (Supporting Information Fig. 2).

### Gd-enhancement pattern

Gd-enhancement was obtained from few samples, as an additional readout pertaining to vascular integrity, with signal enhancement indicating accumulation of contrast agent due to vascular leakage. Preliminary qualitative assessment suggests that A549 grafts display a generally more prominent enhancement in all three samples studied, while only one MC-38 graft displayed similar overall signal enhancement (Supplementary Information Fig. 1C).

Notably, upon visual inspection of the graft and attached CAM after extraction from the egg no difference in the vascularization pattern/ vascular bed surrounding the graft was observed between the different graft types (upper left inserts Fig. 1B). Vessel density was significantly higher in A549 grafts compared with MC-38 grafts as assessed by histomorphometry, p = 0.0037, unpaired t test (Fig. 1D). Chicken vessels within human A549 and murine MC-38 grafts were qualitatively confirmed by two lectin stainings (Supporting Information Fig. 3).

### Histological appearance

A549 and MC-38 cell grafts exhibited distinct phenotypes in terms of cellularity and proliferative status. A549 grafts revealed a more granular appearance in H&E staining in contrast to the more densely packed cellular organization seen within MC-38 grafts. Similarly, proliferative cells were more homogenously distributed within the MC-38 grafts, while A549 grafts they were found rather in patches distributed towards the graft surface (Fig. 1B).

## Discussion

In this study, we used quantitative T2* and oxygen-enhanced T1 MRI in a tumor model planted on the chorioallantoic membrane of the chicken embryo. We investigated if these may serve as functional *in ovo* imaging markers when compared between periods of air and hypercapnic-hyperoxia (carbogen) exposure. A549 and MC-38 tumor grafts were studied and compared in terms of their changes in quantitative T2* and T1. Both responded to carbogen gas exposition, however in different ways. The findings indicate that functional gas challenge with carbogen is feasible through gas exchange on the CAM. Moreover, vascular function as well as oxygenation status of different tumor grafts can be assessed in this experimental model.

MR images were obtained from all tumor grafts with good SNR and image quality to depict the grafts on anatomical images and quantitative parametric maps of T2* and T1 values. The two graft types displayed different size and structure as shown in the MRI images, and corroborated by histology.

Quantitative maps of T2* and T1 obtained under basal condition (air) revealed distinct and different distribution of areas with high T2* and T1 values in both graft types. They were larger in the graft core in the MC-38 grafts, while larger T2* and T1 values were distributed more towards the graft/CAM surface of the graft in the A549 grafts. This topography might be related to the underlying tumor structure: H&E staining revealed a more homogenous, densely packed cellularity for MC-38 grafts, while A549 grafts displayed a more granular cellularity.

Similarly, response to the hypercapnic-hyperoxic gas challenge with carbogen was heterogeneous in the grafts. A graphical, rather simplified approach to compare the two tumor grafts was performed by ROI analysis comprising the whole graft. While thus averaging across heterogeneous graft regions, it was used as a first step towards a more quantitative analysis comparing the two different graft groups, given the differences in graft size. Here, a distinct pattern of responses was observed between the two graft types: A549 grafts displayed a strong response in T2*, with significantly increased T2* values upon carbogen challenge, but no consistent trend in T1. MC-38 grafts in contrast, revealed no consistent response in T1 and T2*. Such different response patterns indicate that the grafts differ in their vascular response. Notably, we also assessed contrast enhancement with Gd-DOTA in a few samples, which appeared more prominent in the smaller A549 grafts. Such enhancement pattern might be supportive for our notion that A549 grafts display a better vascular response (significantly increased T2*). This is supported by a higher vessel density found for A549 grafts compared with MC-38 grafts. In addition, MC-38 grafts exhibited a significantly higher HIF-1-α positive cell density, which might be a result of the lower vessel density, especially in the center of the MC-38 tumors. It has to be emphasized, however, that the total cell density is visibly higher in MC-38 grafts compared to A549 grafts, which relativates the findings of higher HIF-1-α positive cell density.

Although we demonstrate feasibility of this gas challenge approach, there are limitations. Anesthesia represents a prominent confound to any vascular functional study^30,31^ and has been extensively discussed in the context of preclinical functional MRI^32–36^. Vasoreactivity is a tightly controlled process, which is regulated through neuronal, hormonal and metabolic factors. Volatile anesthetics decrease endothelium-dependent vasorelaxation through the nitric oxide signaling pathway, but also injectable anesthetics have been shown to inhibit vasoconstriction to various degrees. Our anesthesia protocol, optimized in our lab previously for the chicken embryo *in ovo*^37^, might also affect our observations with regards to changes in T2* and T1. However, imaging requires immobilization of the chicken embryo and corresponding confounding effects cannot entirely be precluded. Previous studies describe other anesthesia protocols in the context of MRI monitoring of chick development *in ovo* using MRI with a focus on their robustness, repeatability and teratogenic effects in different applications^38–41^, while hemodynamic and metabolic effects have also been discussed^42–44^. An age-adapted cooling regime has been introduced as a viable alternative to pharmacological anesthesia for immobilization of the chicken embryo^18,45–47^.

Sensitivity of the technique is certainly another limitation. While our tumors grafts were 2.6 mm (A549) and 4 mm (MC-38) in diameter, corresponding to a (spherical) volume of about 9 and 33 mm^3^, respectively, the smallest tumor we were able to analyze was 1 mm in diameter (0.5 mm^3^). This limit will allow for some significant reduction in tumor volume, e.g. by treatment, that could still be assessed. Higher field strength MRI systems with optimized coils and faster gradients systems may provide more favorable sensitivity/spatial resolution.

Another aspect pertains to the CAM model itself. Though it represents a versatile, accessible and easy to handle, cheap model, its immunodeficient phase is restricted only to ID 14. Nevertheless, tumors grown within the time window of ID 7 and ID 14 can be well detected and characterized by MRI. After ID 15 the immune system is activated, affecting graft acceptance of the host. In addition, dependent on applicable jurisdiction, experiments may be regarded as full animal experiment from ID15, with all necessary ethical license approvals in place.

The sequential design of the gas challenges, starting under air and continuing with the functional gas challenge, may also be critical. In some samples, repeat measurements under air after the functional challenge revealed drop towards basal levels. Therefore, we adapted our protocol with a 5-minute break between each gas change. The necessity to wait long enough between exposure to different gas challenges has to be taken into account.

Finally, we report upon gross phenotypical histological markers, H&E and Ki-67, to explore the underlying cellular structure and composition of the studied cell grafts. Larger size of MC-38 grafts may be related to its more homogenously distributed and larger number of proliferative cells as observed under Ki-67 staining. In A549 grafts proliferative cells show a more patchy distribution, clustering more towards the surface of the graft. As to whether these phenotypically different cellular distributions contribute to different basal T2* or T1 values in the two graft types, and their contribution to different response to the functional challenge remain to be studied.

## Conclusions

Our proof-of-principle study demonstrates that different tumor grafts planted on the CAM of the chicken embryo display distinct phenotypes that can be distinguished and characterized non-invasively *in ovo* using MRI. Importantly, we show that functional gas challenge is feasible through the CAM and affects MRI signals associated with vascular reactivity and oxygenation status of the graft. This technique allows a non-destructive and easy assessment of different drugs and may be useful for the development of novel cancer models. With the CAM as an intermediary between cell cultures and experimental animal models, and comparative histology readily available, this model of tumor characterization will help to learn more about the underlying functional MRI signal changes that serve as MRI markers for different tumor vascular functional and oxygenation phenotypes. In addition, biopsies from human cancer tissue may be planted onto the CAM and characterized by gas challenge via MRI. As such, our study paves the way for future drug screening and clinical applications where functional gas challenge may help to characterize tumors.

## Supplementary information

Supporting Information.
