# Supplementary material for: Tumor grafts grown on the chicken chorioallantoic membrane are distinctively characterized by MRI under functional gas challenge
Source: Sci Rep. 2020 May 5;10:7505. doi: 10.1038/s41598-020-64290-z (PMC7200801; doi:10.1038/s41598-020-64290-z)
Supplement: Supplementary file 1 — Supporting Information. [file 41598_2020_64290_MOESM1_ESM.pdf]

Supporting Information

Tumor grafts grown on the chicken chorioallantoic membrane are distinctively characterized by MRI under functional gas challenge

Conny F. Waschkies[1], Fatma Kivrak Pfiffner[2], Dorothea M. Heuberger[3], Marcel A. Schneider[4], Yinghua Tian[4], Petra Wolint[5], Maurizio Calcagni[5], Pietro Giovanoli[5], Johanna Buschmann[5]

[1]Center for Surgical Research, University Hospital Zurich, Switzerland

[2]Institute of Medical Molecular Genetics, University of Zurich, Switzerland

[3]Institute of Intensive Care Medicine, University Hospital Zurich, Switzerland

[4]Visceral and Transplant Surgery, University Hospital Zurich, Switzerland

[5]Plastic Surgery and Hand Surgery, University Hospital Zurich, Switzerland

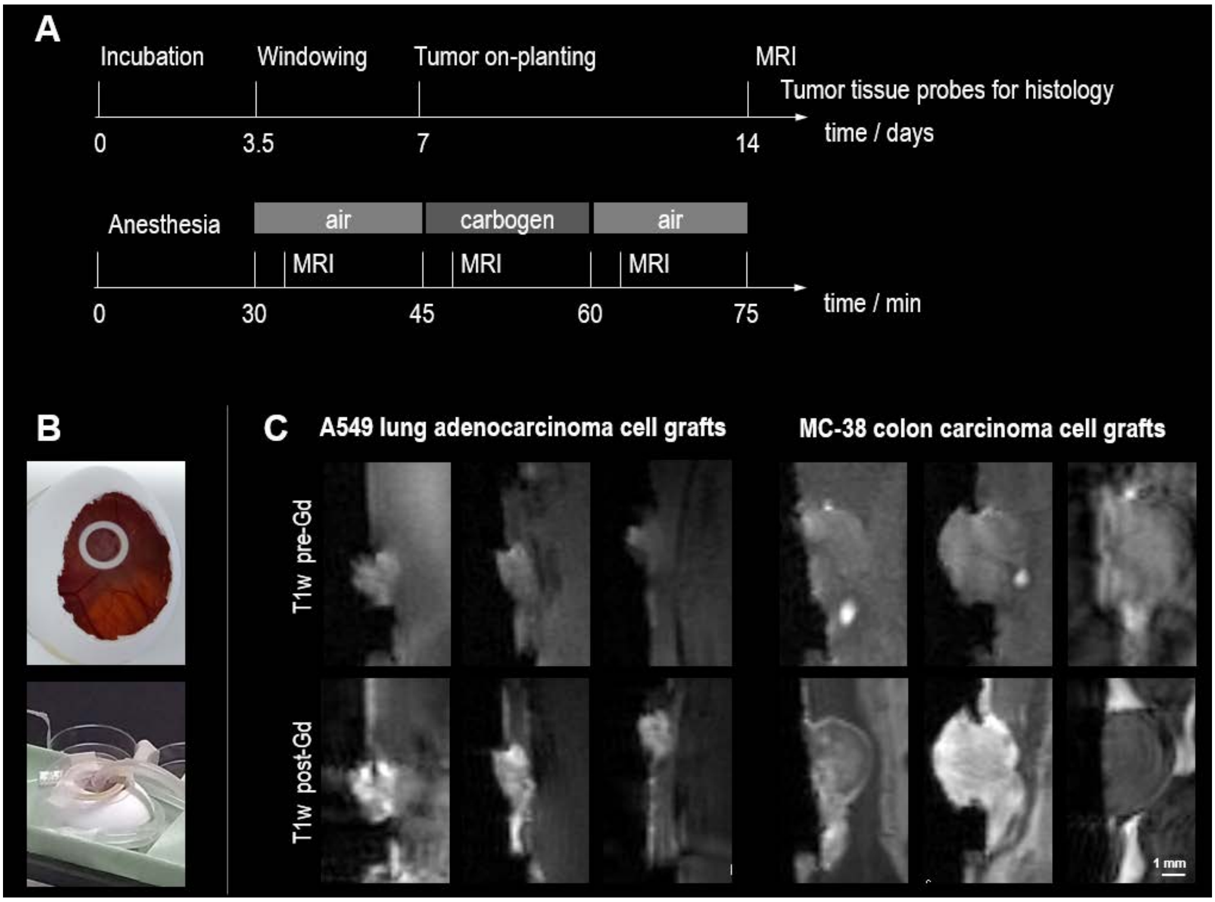

Supplementary Figure 1: A. Experimental schedule. Top: Preparation of eggs for tumor on-planting and MRI. Bottom: Anesthesia with medetomidine and MRI assessment of quantitative T1 and T2* during air and carbogen exposure. 5 min adaptation periods were allowed for each gas change (double lines). **B. Experimental setup.** Top: Tumor cells planted into the middle of a supportive plastic ring on top of the CAM 7 days after grafting. Bottom: Experimental setup with gas challenge delivery tube and surface coil placed over the egg shell window. **C. Gd-enhancement in some of the grafts.** Shown are T1w anatomical images of the grafts *in situ* obtained pre- and post-Gd (15 min after i.v. injection of 100uL Gd-DOTA, Dotarem, Guerbet S.A., Switzerland). Qualitative assessment in the few samples suggests that A549 grafts display a more prominent enhancement compared with MC-38 grafts.

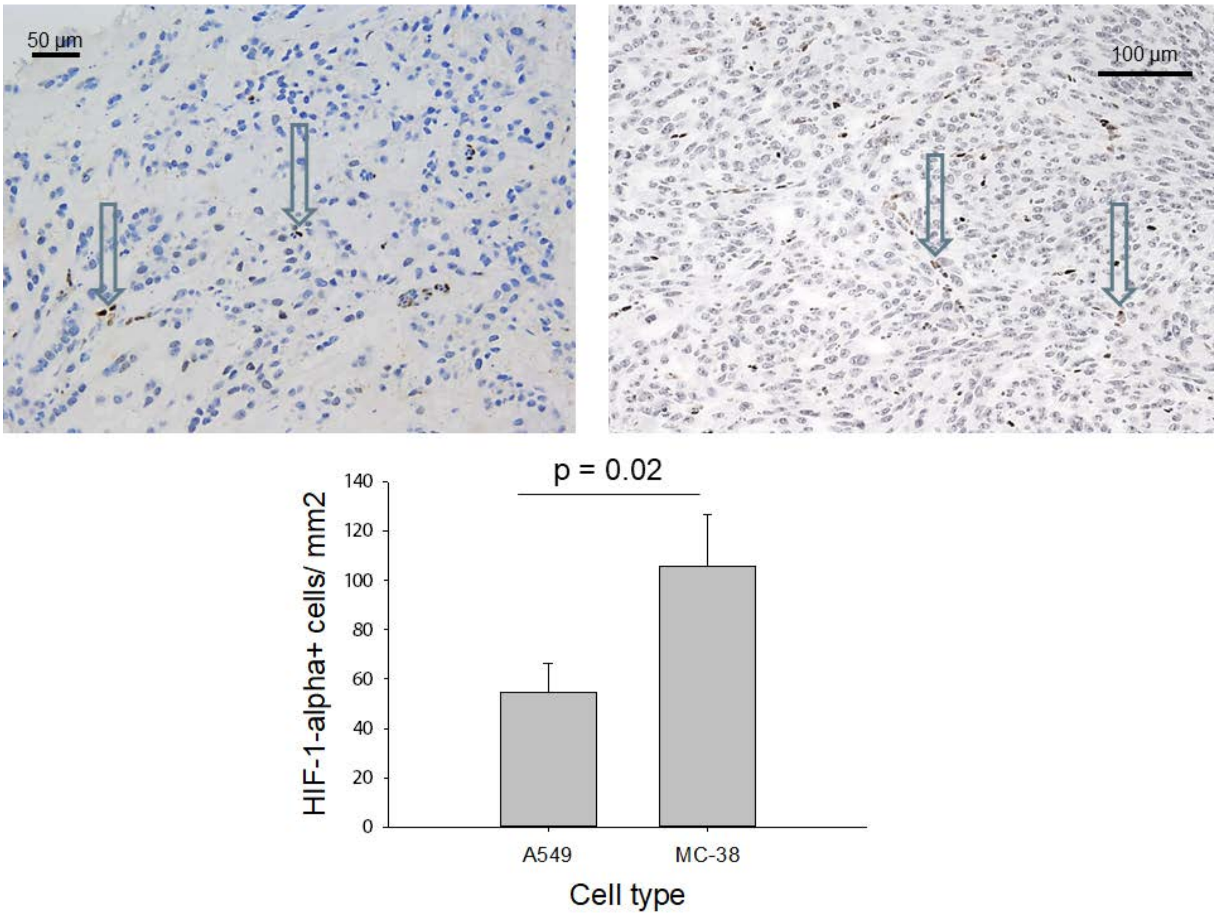

Supplementary Figure 2: Hypoxia assessment. Top: Representative immunohistochemically stained sections of tumors A549 (left) and MC-38 (right) stained for HIF-1-α at magnification 200x. HIF-1-α positive cells appear brown (arrows for exemplary cells), while HIF-1-α negative cells appear blue. Bottom: Quantitative analysis of HIF-1-α positive cells per area for the two cell types under view from n=5 randomly selected tumor grafts of both cell types.

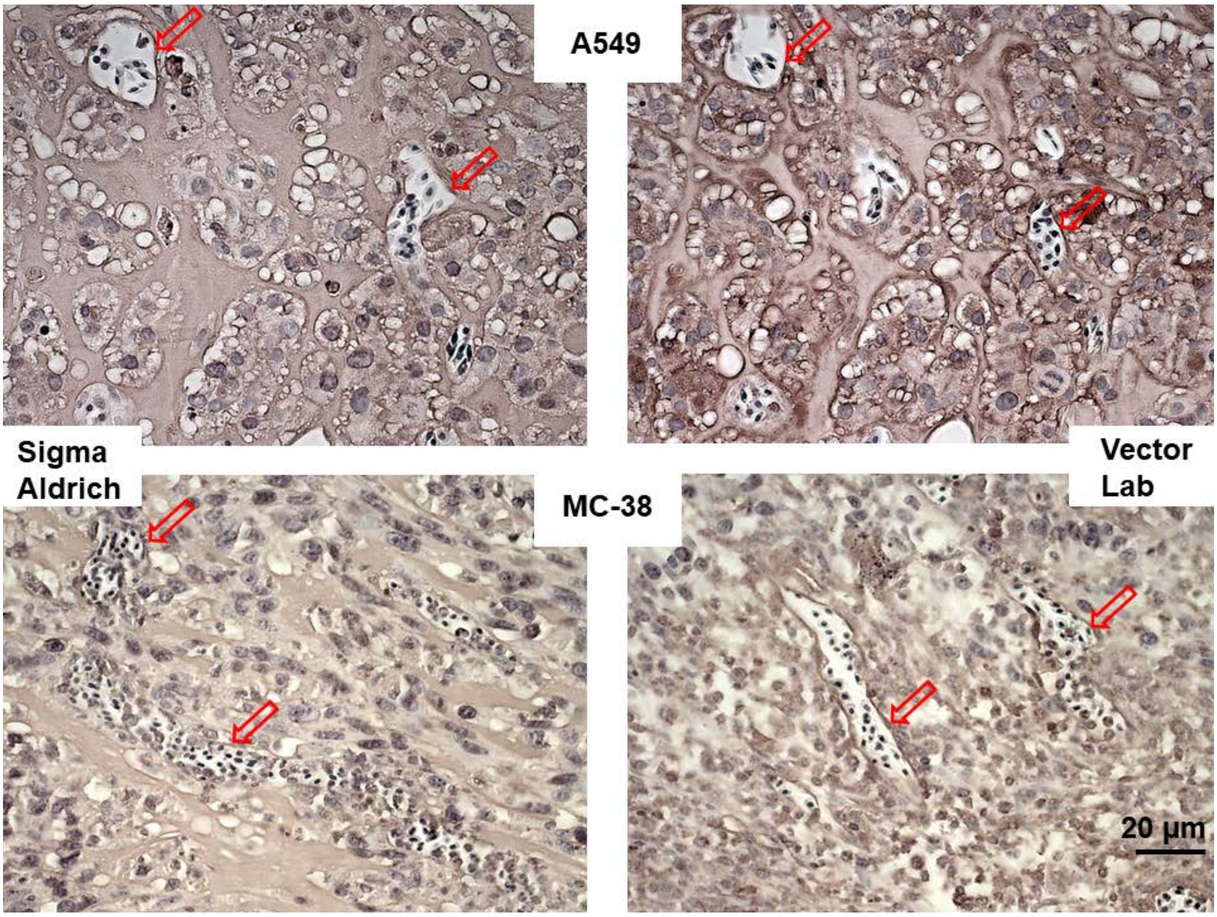

Supplementary Figure 3: Staining of chicken vessels within tumors by lectins. Top: A549 lectin staining with Sigma Aldrich (left) and Vector lab lectin (right). Bottom: MC-38 lectin staining with Sigma Aldrich (left) and Vector lab lectin (right). The luminal side of the chicken vessels is stained dark brown with the lectins. The erythrocytes of the chicken embryos are dark blue, exhibiting a nucleus typical for avian species. Exemplary vessels are depicted with red arrows. Magnification 400x.

Supplementary Table 1: Differences between the two graft types used in this study

Criteria	*A549*	*MC-38*
**Species**	human	murine
**Organ**	alveolar cancer cells	colon cancer cells
**Structure/ anatomy**	granular cellularity	densely packed cellularity
**Size**	approx. 2.5 mm diameter	approx. 4.0 mm diameter
